# Using genomics to better define high-risk MGUS/SMM patients

**DOI:** 10.18632/oncotarget.26390

**Published:** 2018-11-27

**Authors:** Ankit K. Dutta, Duncan R. Hewett, J. Lynn Fink, Andrew C.W. Zannettino

**Affiliations:** Andrew C.W. Zannettino: Myeloma Research Laboratory, Adelaide Medical School, Faculty of Health and Medical Sciences, The University of Adelaide, Adelaide, SA, Australia; Cancer Theme, South Australian Health and Medical Research Institute (SAHMRI), Adelaide, SA, Australia

**Keywords:** multiple myeloma, smoldering multiple myeloma, genomics, intraclonal heterogeneity, clonal evolution

Multiple myeloma (MM) is a haematological malignancy characterised by the clonal proliferation of aberrant plasma cells (PCs) in the bone marrow. The development of novel therapies has seen significant improvements in depth of response, progression-free survival and overall survival of patients. In almost all cases, MM is preceded by asymptomatic disease stages known as monoclonal gammopathy of undetermined significance (MGUS) and smouldering MM (SMM). Currently, patients with MGUS and SMM are not treated until they display one or more of the hallmarks of active disease [1].

The application of next generation sequencing (NGS) to analyse the genetic landscape of patients with MGUS, SMM and MM has revolutionised our understanding of the aetiology and genetic mechanisms of MM initiation and progression. Initial studies focused on the examination of samples isolated from unmatched patients at MGUS, SMM and MM revealing the existence of intraclonal heterogeneity and “Darwinian” evolution as a hallmark of MM [2–5]. Furthermore, significantly mutated genes were identified including KRAS, NRAS, BRAF, TP53, DIS3 and FAM46C, which are believed to be drivers of MM due to their recurrent nature [2, 4, 5]. The acquisition of driver mutations is thought to confer an improved clonal fitness, thereby allowing PC clones to survive and progress to MM.

Subsequent studies examined the genetic changes associated with tumour evolution and transformation from MGUS/SMM to MM. **These studies analysed rare matched patient samples isolated from the same patient when first diagnosed with MGUS or SMM, and subsequently with MM [5–8]**. Initially, two studies investigated the tumour evolution associated with the progression of SMM to MM *(n* = 4) [5, 8]. These studies identified that the majority of genetic changes present at the MM stage were already present at the asymptomatic SMM stage. These findings suggest that the progression of SMM to MM does not require the acquisition of new mutations, but is associated with changes in the relative proportions of distinct clones that make up the tumour, a phenomenon termed “clonal progression” [5]. While these studies characterised the intraclonal heterogeneity present from the SMM stage, the subclonal tumour evolution model associated with disease progression was poorly defined.

Two recent studies have definitively catalogued the genetic changes and clonal evolution that accompany the progression from MGUS/SMM to MM [7], and SMM to MM [6], using larger numbers of paired samples and two different NGS analyses methods. We recently performed whole exome sequencing (WES) analyses of paired MGUS-MM (*n* = 5) and SMM-MM (*n* = 5) samples, finding that progression to MM is characterised by “clonal stability”. This is where the transformed subclones identified at MM were found to be present at the MGUS/SMM stages, with progression associated with subtle changes in the emergence or extinction of child subclonal populations [7]. In contrast, Bolli et. al. performed whole genome sequencing (WGS) analyses of SMM-MM (*n* = 10) and identified two models of evolution, namely “static progression” (*n* = 4) and “spontaneous evolution” (*n* = 6) [6]. Similar to “clonal stability”, “static progression” describes a model in which there is limited, or no significant, changes in subclonal architecture between SMM and MM stages. Conversely, the “spontaneous evolution” model defined a branching architecture, where the acquisition of mutations within individual subclonal populations conferred a selective advantage for survival. Notably, both studies found “clonal stability”/”static progression” to be associated with a short time to progression (MGUS-MM: median ∼38 months [7]; SMM-MM: median ∼14 months [7] and 5.5 months [6]) compared to the established median of up to 25 years or greater for MGUS to MM, and less than 5 years for SMM to MM [5]. Interestingly, Bolli et. al. demonstrated that “spontaneous evolution” was associated with a longer time to progression (median 23 months), possibly reflecting the time required for a clone to acquire sufficient genetic change to confer a growth advantage [6]. Collectively, these studies suggest that MGUS/SMM patients who progress in a short time frame possess subclones harbouring the required mutations to be on the threshold of transformation to MM.

MGUS and SMM represent largely asymptomatic conditions that with current standard-of-care remain untreated. Instead a careful “watch and wait” strategy is employed in which patients are monitored for signs of MM progression. These new NGS studies illustrate that PCs from rapidly progressing MGUS/SMM patients can be as genetically and clonally complex as those of MM patients. These findings highlight the need to redefine these asymptomatic patients beyond clinical criteria to include genomic data, which can be used to provide a “genomic risk of progression” measure to determine which pattern of progression they may undertake. This approach would enable the selection of patients for whom early treatment would prevent disease progression. This is especially important for SMM, which is a heterogeneous intermediate disease stage between MGUS and MM where two subsets of patients have been identified; one group exhibits indolent disease akin to MGUS, while another group displays aggressive disease course like MM. Consequently, being able to define these high-risk “MM like” SMM patients, who are yet to progress, with the addition of genomic biomarkers will allow treatment strategies to prevent onset of MM. Notably, recent NGS studies examining high-risk SMM patients (*n* = 186), suggest that this group displays a higher mutational load (1.44 mutations/Mb), compared to low-risk SMM patients (0.73 mutations/Mb), and mutations in the MAPK and NFkB pathways [9]. This high mutational load is comparable to that of the median somatic mutation rate of MM, which is observed to be 1.6 mutations/Mb. Indeed, the first clinical trial (QuiRedex) examining the value of early treatment of high risk SMM patients, using induction therapies lenalidomide and dexamethasone, has shown significant survival advantage of patients treated early (vs. standard of care monitoring) with a prolonged overall survival (3-year survival rate of 94% vs. 80%) [10].

Further large cohort studies of paired samples from both low-risk and high-risk patient populations will be required to identify the specific genomic biomarkers that are associated with each of the progression models described. Ultimately, the integration of genomic data into a new risk stratification paradigm will provide clinicians with better tools to determine which, and when, a patient should be treated.
